# A realistic implementation of ultrasound imaging as a human-machine interface for upper-limb amputees

**DOI:** 10.3389/fnbot.2013.00017

**Published:** 2013-10-22

**Authors:** David Sierra González, Claudio Castellini

**Affiliations:** Robotics and Mechatronics Center, German Aerospace Center (DLR)Weßling, Germany

**Keywords:** ultrasound imaging, human-machine interaction, human-machine interfaces, rehabilitation robotics, force control, incremental learning

## Abstract

In the past years, especially with the advent of multi-fingered hand prostheses, the rehabilitation robotics community has tried to improve the use of human-machine interfaces to reliably control mechanical artifacts with many degrees of freedom. Ideally, the control schema should be intuitive and reliable, and the calibration (training) short and flexible. This work focuses on medical ultrasound imaging as such an interface. Medical ultrasound imaging is rich in information, fast, widespread, relatively cheap and provides high temporal/spatial resolution; moreover, it is harmless. We already showed that a linear relationship exists between ultrasound image features of the human forearm and the hand kinematic configuration; here we demonstrate that such a relationship also exists between similar features and fingertip forces. An experiment with 10 participants shows that a very fast data collection, namely of zero and maximum forces only and using no force sensors, suffices to train a system that predicts intermediate force values spanning a range of about 20 N per finger with average errors in the range 10–15%. This training approach, in which the ground truth is limited to an “on-off” visual stimulus, constitutes a realistic scenario and we claim that it could be equally used by intact subjects and amputees. The linearity of the relationship between images and forces is furthermore exploited to build an incremental learning system that works online and can be retrained on demand by the human subject. We expect this system to be able in principle to reconstruct an amputee's imaginary limb, and act as a sensible improvement of, e.g., mirror therapy, in the treatment of phantom-limb pain.

## 1. Introduction

The term *ultrasound* is used to refer to sound (pressure) waves of frequency over 20 kHz. These sound waves are routinely produced by natural phenomena as well as some animal species such as, e.g., bats to navigate flight and to locate food sources. In the 40 s and 50 s (Dussik, 1942; Donald et al., 1958) it was discovered that, thanks to their capability of penetrating the soft tissues without harming them, focussed ultrasound waves could be employed to visualize the innards of the human body and used as a diagnostic tool. The technique has turned out to be so powerful and useful that today medical ultrasound imaging (also known as medical ultrasonography, hereafter US imaging) is routinely used in hospitals for diagnostic purposes.

**Figure 1:**
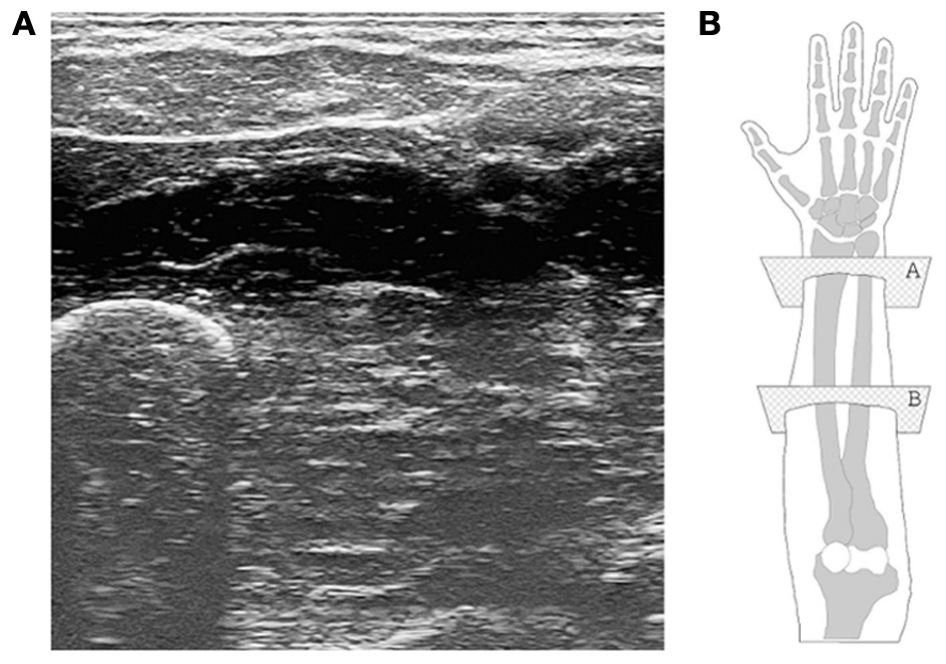
**(A)** A typical ultrasound image obtained during the experiment. The ulna is clearly visible in the bottom-left corner, while the flexor muscles and tendons are seen in the upper part. **(B)** A graphical representation of the human hand and forearm (right forearm; dorsal side up). The transducer is placed onto the *ventral* side; plane “B” corresponds to the section from which the ultrasound image was taken.

Modern US imaging (Cobbold, 2007) fully exploits the principle of wave reflection and advanced microelectronics to obtain two- or three-dimensional live images of the body parts of interest. An array of piezoelectric transducers generates a multiplexed, focused beam of ultrasound waves which penetrates the body part; partial reflection of the waves at the interfaces between tissues with different acoustic impedance is then converted to a gray-scale 2D image. High values of gray denote tissue interfaces. Modern US imaging machines are portable or even hand-held and can achieve sub-millimeter spatial resolution and 100 Hz temporal resolution, penetrating several centimeters below the subject's skin (Jensen, 2002). US imaging has no known side effects (World Health Organisation, 1998) and is routinely used in most hospitals. Figure 1 shows a typical ultrasound image, obtained from a human forearm.

US imaging is widely used to detect conditions of the musculoskeletal system (Kane et al., 2004) and carries a good deal of information about the configuration of the human hand. Such a wealth of information is beginning to be exploited to build a novel Human-Machine Interface (HMI) with clear future applications in, for instance, advanced hand prosthetics, and potential to become a serious competitor to more established non-invasive peripheral-nervous-system-machine interfaces such as, e.g., surface electromyography. Recently, extensive work by Zheng et al. (2006); Chen et al. (2010); Jing-Yi et al. (2011) and ourselves (Castellini and Passig, 2011; Castellini et al., 2012) has revealed that US imaging can actually be used as an HMI. In Castellini and Passig (2011) in particular, we have for the first time shown that a linear relationship exists between the angles at the metacarpophalangeal joints of the human hand and spatial first-order features extracted from US images of the forearm. Since the metacarpophalangeal joints are those at the basis of the fingers (linking each finger to the palm), it is possible to reconstruct the hand configuration to a good degree of precision, using the US images of the forearm. The information extracted using such features is positional, allowing the system to work irrespective of the velocity of the subject's movement, the only limitation lying in the hardware and software (i.e., the imaging rate of the ultrasound machine, the computational power, etc.).

Previous work with surface electromyography (Castellini et al., 2009; Tenore et al., 2009) shows that a remarkable residual activity is present in trans-radial amputees even decades after the operation; therefore we hypothesize that US imaging, which is far more detailed than electromyography, could effectively be used to visualize the imaginary limb of an amputee, or of a nerve-injury patient. The main application of such an achievement would be that of rehabilitating patients whose motor function is impaired, by showing them what they actually desire to do. An even more interesting idea is that of treating phantom-limb pain and other forms of neuropathic pain, for instance complex regional pain syndrome (CRPS): the (albeit partial) restoration of the broken sensorimotor feedback loop might have beneficial effects on it, since mirrored, imagined and executed movements of the phantom limb are known to be negatively correlated to phantom-limb pain (Ramachandran et al., 1995; Chan et al., 2007; Diers et al., 2010).

In the ideal case the system should be extremely lightweight and easy to use; in particular, the calibration phase (training) *must be quick, cannot involve sensors, and must only involve very simple tasks*: amputees and CRPS patients can usually control their imaginary limbs only to a very limited degree of dexterity, such as, e.g., imagining to flex or extend a finger; but it is very unlikely that they can perform graded tasks, as they have no actual cognition of the position/force they are applying. A further requirement is that of being able to add new knowledge as the patient requires it; that is, *the system must work incrementally*: it must be bounded in space and fast, and it must allow for fast retraining whenever required.

In this paper we move along this line, proposing a detailed analysis of the possibilities given by US imaging as an HMI, and using it in a realistic way, according to the above requirements.

First of all, we shift the focus from joint angle prediction to *finger force* prediction. From the point of view of prosthetic applications, this enables force/impedance control as opposed to position control, allowing for a more natural, dexterous interaction with the environment and the objects to be grasped and manipulated. Secondly, we show how a system based upon simple linear regression can be tuned to fulfill the above requirements: we show that a linear relationship exists between spatial first-order US image features and forces at the fingertips; we show that it suffices to gather data from a human subject *only when resting and exerting maximum force*, and the model will then be able to correctly predict the intermediate force values, too, to an acceptable degree of precision; we show that no force sensor is required to train the system: a visual stimulus can be directly used as the ground truth, therefore relieving the patient from using additional hardware - the data collection can be reduced to *pressing one's fingers on a table*. Lastly, we show that the system can be re-calibrated each time a new US image is available, keeping the prediction speed at cinema quality (30 Hz). We first analyse how the prediction error changes as new samples are taken into account; we then perform an online experiment showing that, as the prediction degrades due to external factors (in this case, a shift in the position of the ultrasound transducer), the system can acquire new knowledge and incorporate it, restoring the previous prediction accuracy.

This work is an extension and a completion of Castellini and González (2013).

## 2. Materials and methods

### 2.1. Participants

Ten healthy human subjects (ages 28.5 ± 4.86, max 40, min 23, all right-handed, gender: 9 males, 1 female) joined the experiment. Each subject received a thorough description of the experiment, both in oral and written form. Informed written consent was obtained from all participants. Experiments with ultrasound imaging were approved by the Ethical Committee of the DLR.

### 2.2. Experimental setup

#### 2.2.1. Ultrasound imaging

Ultrasound images were gathered using a General Electric *Logiq-e* portable ultrasound machine equipped with a 12L-RS linear transducer (also called *probe*). The machine was set to B-mode, resulting in a gray-valued image representing a section of what lies directly under the probe, and configured with the following settings: ultrasound frequency of 12 MHz, edge enhancement on, focus point at a depth of about 1.3 cm, minimum depth of field. This results in a frame rate of 38 Hz.

**Figure 2:**
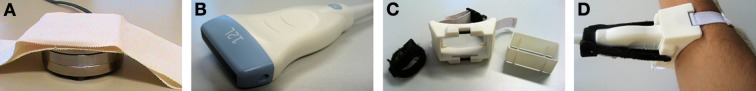
**Parts of the setup. (A)** ATI Mini45 force sensor, fixed to the table. The subjects press on its top; **(B)** linear ultrasound transducer GE 12L-RS; **(C)** custom-made transducer cradle, disassembled; **(D)** transducer attached onto a subject's forearm, using the cradle.

Movement of the probe with respect to the subject's skin, which would have severely hampered the system [see Castellini et al. (2012) again] was avoided using a custom-built plastic cradle obtained via rapid prototyping. The cradle hosts the transducer's head on one side (velcro straps attach the transducer to the cradle), while being lightly but firmly tied to the forearm on the other side by means of a biocompatible elastic band and a side-release buckle. Figures 2B–D shows the transducer, the cradle and the combination of the two fixed on a subject's forearm.

After extensive initial visual checks, we fixed the transducer on the ventral side of the forearm, at a distance of about 10 cm from the elbow. The typical output image (consider Figure 1 again) contains the ulna and the main flexor muscles and tendons. The images are captured from the ultrasound machine's VGA video output using a commercial PCIe video capture card, running at 60 frames per second. As the frames are captured asynchronously with respect to the ultrasound machine, not all of them are whole ultrasound images. In order to avoid considering torn or repeated frames, we enforced the same kind of filtering of Castellini et al. (2012), obtaining a *valid* frame rate of slightly less than 30 frames per second.

#### 2.2.2. Fingertip forces

A single ATI Mini45 SI-290-10 force sensor was employed to capture the force exerted by each finger in turn. This sensor features a guaranteed linear output and a resolution of $\frac{1}{8}\text{N}$. The sensor was taped onto the setup table at a convenient distance from the subject's hand, so that a minimal movement would be involved in pressing it with each finger. The sensor was connected to a DAQ card, and its values were streamed over UDP onto the local network. Figure 2A shows the force sensor.

### 2.3. Experimental protocol

The main experiment of this study consisted of data collection only; US images, force readings from the sensor and stimulus values were synchronously recorded. Section 3 describes a second experiment in which data were captured and processed online. The second experiment closely follow the guidelines of the main one.

**Figure 3:**
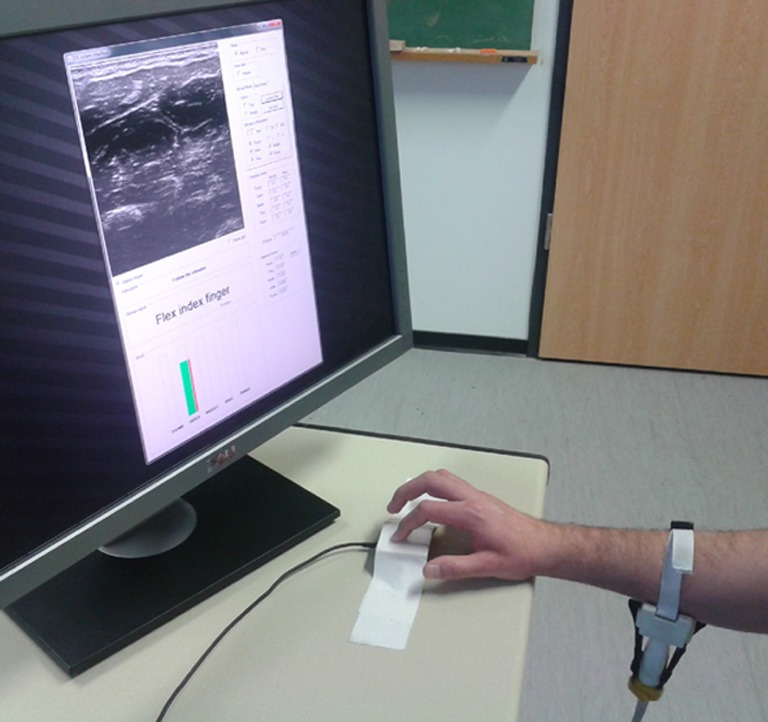
**A bird's eye view of the setup.** The subject sits in front of a screen on which the stimulus is shown; meanwhile force data and US images are recorded.

At the beginning of the experiment, each subject sat comfortably on an adjustable office chair, maintaining an upright body posture with both feet on the floor and the elbow bent at 90°. Certified US gel was applied directly to the skin over the target area, approximately 10 cm below the elbow. The US probe was then fixed to the forearm using the custom cradle. In front of the subject, and directly next to the force sensor, a computer screen showed the live US images and the experiment instructions. Figure 3 shows a bird's eye view of the setup.

Initially each subject was asked to press the sensor once with each finger, applying the largest possible force without feeling discomfort or pain. This way we gathered an indication, *F*_*max*_, of the maximum forces applicable by each subject at each finger. The subjects were then asked to simply lean their dominant hand on the table next to the sensor and, during the experiment, do as instructed by a visual stimulus.

**Figure 4:**
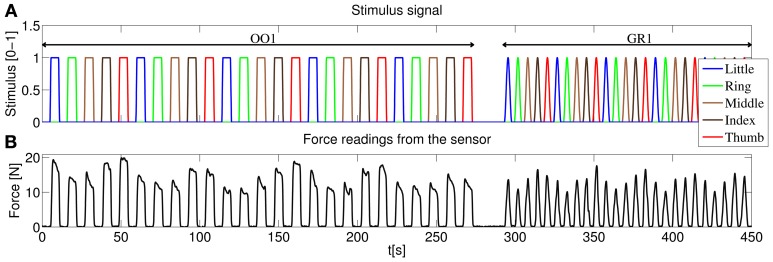
**(A)** Structure of the stimulus shown to the subjects, first session. In the on-off phase (OO1), only rest and maximum force are induced for each finger, each repetition consisting of 4.5 s of force application, followed by 4.5 s of rest. Five repetitions per finger are induced. In the graded phase (GR1) the subjects must exert force following a squared sinusoidal pattern. **(B)** Forces as measured by the force sensor during the experiment for a typical subject.

The experiment consisted of two identical sessions, and each session was likewise divided in two parts, according to the kind of stimulus administered: an *on-off* phase (OO) and a *graded* phase (GR). The complete structure of the stimulus for one of the sessions is displayed in Figure 4A. The different phases will be hereafter denoted as OO1 and GR1 (for session 1) and OO2 and GR2 (for session 2).

#### 2.3.1. On-off phase

During these phases (OO1 and OO2), the stimulus induced the subject to either rest or apply maximum force with each finger. The subject was presented with both text banners (e.g. “rest”, “press with the index finger” or “press with the thumb” - notice that pressing with the thumb in this configuration is tantamount to rotating it, for example when hitting a piano key with the thumb) and five green wide vertical bars, one for each finger, that got filled whenever the subject had to apply force. The subjects were instructed to press with the required finger on the sensor applying “a reasonably large amount of force.” This intentionally fuzzy indication reflects what can be asked of an amputee.

As depicted in Figure 4A during the on-off phase the subject was told to rest or apply force with each finger in turn (little, ring, middle, index and thumb), and the whole cycle was repeated 5 times. Each flexion lasted 4.5 s, and 4.5 s of rest were allowed in-between flexions. Additionally, the transition of the vertical green bars from rest to maximum force and vice versa lasted 1 s each. This results in a duration of 5 × 5 × (4.5 s + 1 s + 4.5 s + 1 s) = 275 s for each on-off phase.

#### 2.3.2. Graded phase

In the graded phase, the subject was induced to exert forces following a squared sinusoidal pattern, i.e., to apply a full range of forces from none to maximum. During this phase *two* colored vertical bars were displayed on the screen: a wide green bar representing the required force and a narrow red bar showing the force actually applied at the sensor's surface. The stimulus for the required force was chosen as 0.8*F*_*max*_sin^2^(*t*). Figure 4B shows the force measurements of the sensor for a typical subject during one of the sessions. In this case each pattern (from rest, increasing the force to maximum then decreasing again to rest according to the sin^2^ pattern) lasted 4.5 s, and 1.5 s of rest was allowed in-between flexions. This results in 5 × 5 × (4.5 s + 1.5 s) = 150 s for each graded phase.

#### 2.3.3. Data synchronization

All in all the experiment lasted 275 s + 150 s + 275 s + 150 s = 850 s = 14′10 s. No subjects reported discomfort of fatigue during or after the experiment. US images, force measurements and stimulus values were initially inspected to ensure that no delay was introduced during the UDP transmission of the forces. This allowed us to use the valid frame rate, 30 Hz, as the global sampling frequency. (Notice anyway that the bandwidth of the signals we are interested in, i.e., frames and force data, is directly dependent on the stimulus, that is less than 1 Hz).

### 2.4. Visual features

#### 2.4.1. Feature extraction

From each ultrasound image the same kind of visual local features used in Castellini et al. (2012) were extracted; namely, 181 uniformly distributed circular regions of interest (ROIs) of radius 20 pixels were selected on the image, each ROI center being 50 pixels apart from each other. These values are the optimal trade-off between the required amount of information and computational feasibility, and were determined in an initial round of experiments—this was already determined off-line in the aforementioned paper.

**Figure 5:**
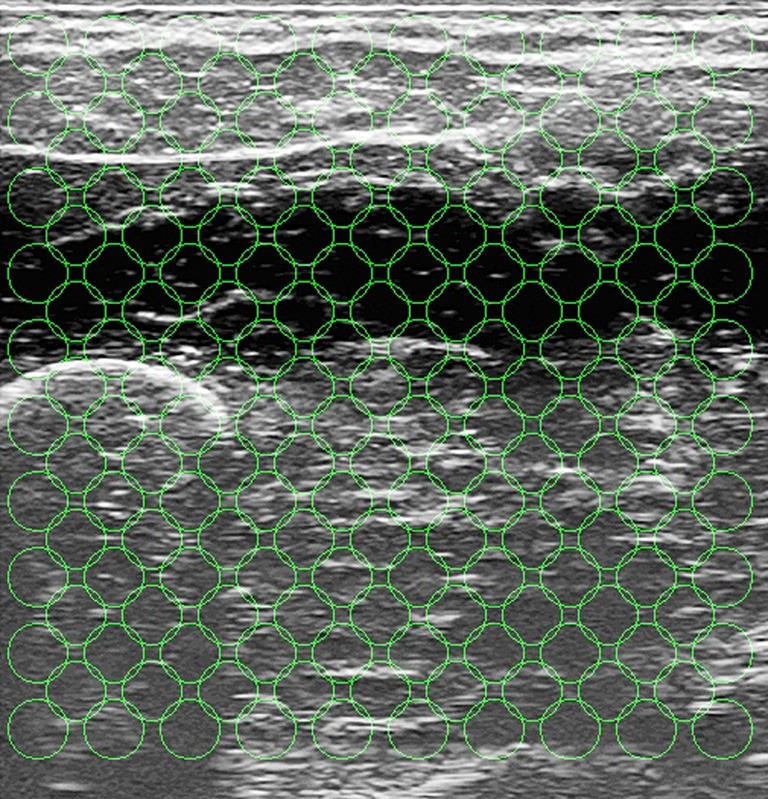
**The grid of ROIs, superimposed to the typical shot seen in Figure 1.** Each ROI has a radius of 20 pixels, and each ROI center is 50 pixels apart from each other.

The motivation for choosing a uniformly-spaced grid is that we are not interested in targeting precise anatomical features projected on the image, but rather to have a thorough although compact representation of the deformations induced by the application of forces. Local spatial approximations are preferred with respect to, e.g., global features (histograms) and temporal derivative features (optical flow) since, as it emerges from visual inspection of the images (Castellini et al., 2012), local changes in the images are related to the anatomical structures involved in the applied forces. For instance, flexing the little finger is enacted by flexing a part of the *M. Flexor Digitorum Superficialis*, whose projection on the ultrasound images (in our setting) is localized in the upper-left corner. The changes in the images *positionally* reflect the movement of the muscle, therefore being related to the exerted forces. Figure 5 shows the grid of ROIs superimposed to the typical shot visible in Figure 1.

More in detail, about the extracted features: let the *i*th ROI be centered around (*x*_*i*_, *y*_*i*_); then from each ROI three real numbers (α_*i*_, β_*i*_, γ_*i*_) were computed, such that the gray values of each pixel (*x*, *y*) ∈ ROI_*i*_ would be approximated by α_*i*_(*x*_*i*_ − *x*) + β_*i*_ (*y*_*i*_ − *y*) + γ_*i*_. Intuitively, α_*i*_ denotes the mean image gray-scale gradient along the *x* direction (rows of the image), β_*i*_ is the same value along the *y* (columns) direction, and γ_*i*_ is an offset. The three features represent a first-order spatial approximation of the gray values of the ROI, accounting for the morphological structure of that region. In order to extract these features (and for all other image-related computations and evaluations) we used the HALCON v10.0 library by MVTec (see www.mvtec.com/halcon). Since three numbers were extracted from each ROI, the dimension of one US sample is 181 · 3 = 543.

Notice that no mechanism compensating the movement of the probe with respect to the subject's skin is here enforced, as it had been done in Castellini et al. (2012). The reasons for this choice are explained in the Discussion Section.

All signals (force and visual features) were lowpass filtered with a Butterworth first-order low-pass filter, cutoff frequency of 1 Hz. From the data in the on-off phases only the last two thirds of the on and off periods were taken into account in order to avoid considering the transitions from rest to maximum force and vice-versa.

#### 2.4.2. Qualitative feature analysis

**Figure 6:**
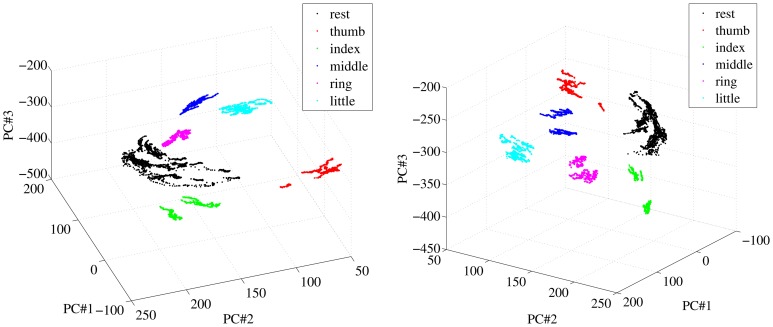
**Two different views of a three-dimensional PCA projection of the samples obtained from a typical subject during OO1 and OO2.** Colors denote finger flexions and rest.

A qualitative analysis of the patterns corresponding to finger forces, as they appear in the input space, was performed initially; in particular, we were interested to determine how different from one another they were. Figure 6 shows two different views of some of the visual feature samples obtained from a typical subject, reduced to three dimensions using Principal Component Analysis. Each color denotes a subset of the features. The samples labeled “rest” are obtained by selecting all samples in the OO1 and OO2 phases corresponding to the last two thirds of each off period; the other sets are obtained in the same way for each finger, but considering instead the last two thirds of each on period.

As is apparent from Figure 6, at least in this case each reduced pattern obtained during extreme forces (minimum and maximum) is clearly clustered and occupies a different portion of the reduced input space. The patterns representing the resting state are all grouped into one single cluster. In order to check whether this property holds in general for our dataset, we have checked, for each subject separately, how separated these clusters are in the original, 543-dimensional space. The chosen measure of separatedness is the following: let *C*_*i*_ and *C*_*j*_ denote two of the aforementioned clusters, and let σ_*i*_ ∈ ℝ^543^ be the standard deviation of *C*_*i*_; then a *safety index s*_*ij*_ is defined between the two clusters as

$$s_{ij}=\frac{\max\{\sigma_{i}\}}{\left||Ci-Cj|\right|}$$

**Figure 7:**
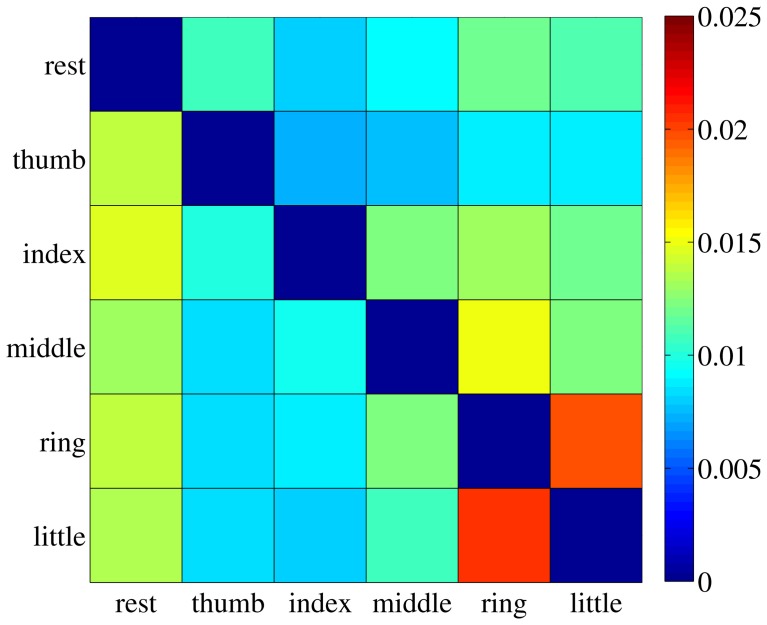
**The general safety matrix.** Each entry of the matrix, *s*_*ij*_, is the safety index between clusters *C*_*i*_ and *C*_*j*_, that is the ratio of the maximal standard deviation of cluster *C*_*i*_ and the Euclidean distance between the two clusters. Values averaged over all subjects.

The value *s*_*ij*_ is therefore the ratio between the maximum over all dimensions of the standard deviation of cluster *C*_*i*_ (the largest width of the cluster), and the Euclidean distance between clusters *C*_*i*_ and *C*_*j*_. A small value of *s*_*ij*_ indicates that most elements in *C*_*i*_ are far away from *C*_*j*_, therefore hinting at a good separability between the two clusters. For each subject the safety index among all pairs of clusters was computed, leading to a *safety matrix S* = {*s*_*ij*_}; we then averaged out all safety matrices, obtaining the general safety matrix, visible in Figure 7.

The highest value in the matrix is 2.054% (little and ring fingers) indicating that in all cases the patterns employed for the on-off training are extremely well separated in the input space and could be effectively classified, if required^1^.

We have also run a standard linear classification method [namely a Support Vector Machine with linear kernel (Boser et al., 1992; Cristianini and Shawe-Taylor, 2000)] on all clusters, subject per subject. The dataset was first shuffled, then training was performed on one tenth of it while testing was done on the remaining 90%; this procedure was repeated 50 times, each time with a different shuffling. The classification error (balanced error rate) is in all cases extremely low, namely, the highest error rate is 2.7% ± 1.8%. Notice that in Shi et al. (2012) a more complex experiment was set up, in which classification recognition rates of finger flexion motions ranged from 92 to 97%.

### 2.5. Approximating finger forces via ridge regression

We hereby try and extend to forces the analysis first described in Castellini et al. (2012), according to which a *linear relationship* exists between the ultrasound image features described in the previous Section and the angles at the metacarpal hand joints. The analysis is performed separately according to which signal is considered the ground truth: either the force, as recorded by the force sensor, or the stimulus. In the first case we consider the force applied by each finger during the flexion, and zero force otherwise; in the second, the stimulus itself is used, with the hope that the subject has followed it with a certain degree of precision. Notice that this second scenario reflects the typical situation with an amputee, in which *no ground truth is available in principle* and one must resort to either imitation learning or bilateral action [see, e.g., Castellini et al. (2009); Nielsen et al. (2011)]—using a visual stimulus and instructing the subjects to imitate it is tantamount to imitation learning.

For each finger and each type of ground truth (force or stimulus values), a linear mapping is determined between the feature vector **v** ∈ ℝ^543^ extracted from each frame and the ground truth value *g* ∈ ℝ: *g* = **w**^*T*^**v**. The feasibility of the linear approximation is checked by considering the square-root mean-square error normalized over the range of the target values (nRMSE), between the ground truth and the predicted values.

#### 2.5.1. Ridge regression

In order to find the optimal **w** ∈ ℝ^543^ we used a standard technique called *ridge regression*, which is a regularized variant of least-squares regression. In general, given *n* (sample,target) pairs {**x**_*i*_, *y*_*i*_}^*n*^_*i* = 1_ as gathered during the data acquisition, the optimal **w** is

$$w={\left(X^{T}X+\lambda I_{d}\right)}^{-1}X^{T}y$$

where the matrix and vector *X*,**y** are formed by juxtaposing all samples and target values, *d* = 543 is the dimension of the input space, *I*_*d*_ is the identical matrix of order *d* and λ > 0 is the regularization coefficient, which we consistently set at the standard value of 1.

#### 2.5.2. Cross validation

Each **w** was evaluated using 10% of the data set under examination (e.g., 10% of one session) chosen at random, then predicting the ground truth values for the remaining 90% of the set. This procedure was repeated 50 times in order to smooth out statistical differences among the sets used for the evaluation.

#### 2.5.3. Complexity

The evaluation of **w** involves inverting a *d* × *d* matrix, therefore the time complexity of ridge regression is dominated by *d* rather than by *n*: its time complexity is 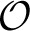
(*d*^3^ + *nd*^2^), its space complexity is 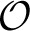
(*d*^2^ + *nd*) and the complexity of a prediction is 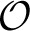
(*d*).

### 2.6. Ultrasound features are linearly related to finger forces

#### 2.6.1. Aim

To determine whether the forces exerted at the fingertips by a healthy human subject can be predicted using a linear combination of the visual features extracted from the US images of the forearm.

#### 2.6.2. Results

**Figure 8:**
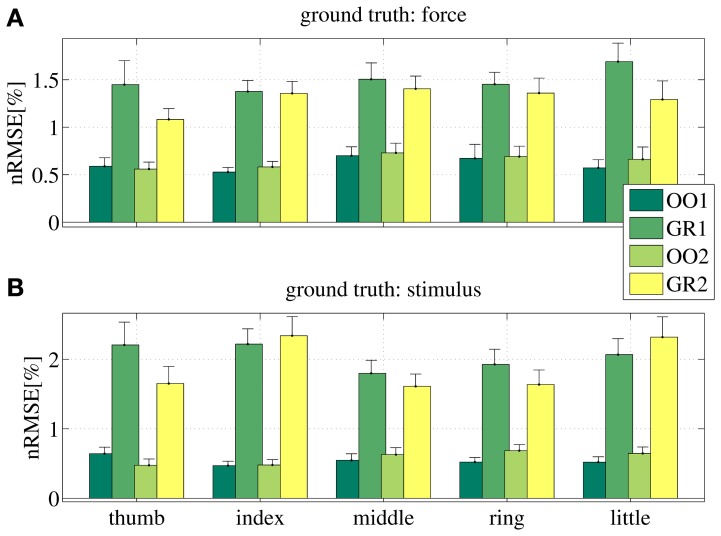
**Normalized root-mean-square error obtained by the linear prediction of force **(A)** and visual stimulus **(B)** for a typical subject, for each session (OO1, GR1, OO2 and GR2) and for each finger.** Each bar and stem represents the mean nRMSE and one standard deviation obtained over the 50 cross-validation folds considered.

**Figure 9:**
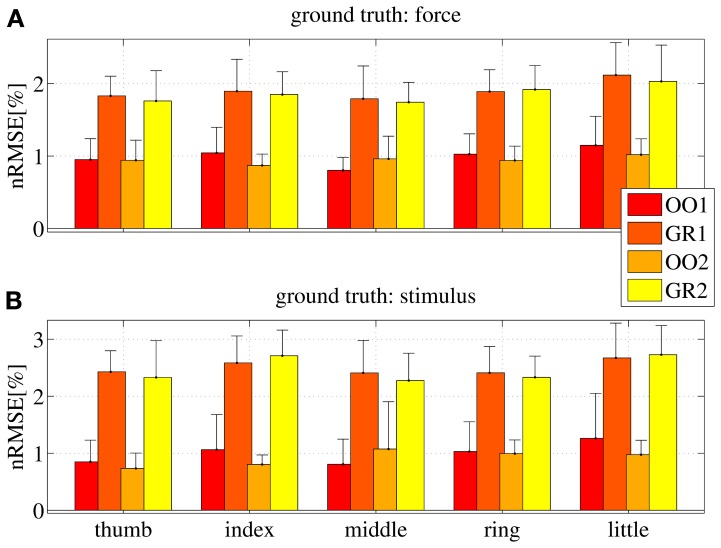
**Normalized root-mean-square error obtained by the linear prediction of force **(A)** and visual stimulus **(B)** for all subjects, for each session (OO1, GR1, OO2 and GR2) and for each finger.** Each bar and stem represents the mean nRMSE and one standard deviation obtained over all subjects.

Figure 8 shows the prediction error for a typical subject, for each session (OO1, GR1, OO2 and GR2) and finger. In (Figure 8A) the force values are used as ground truth, whereas in (Figure 8B) the stimulus values are used. The analysis was repeated for all subjects. Figure 9 shows the error values averaged across all subjects.

As is apparent from the Figures (consider especially Figure 9), the linear regression is able to approximate all required values to a remarkable precision. All on-off values are predicted with a nRMSE of 1% of the force ranges or less. Graded phases (GR1 and GR2) exhibit a higher error, slightly higher than 1.5% in case the force is used as ground truth, and slightly higher than 2% in case the stimulus is used. These results are consistent across subjects and fingers. We believe this is reasonable, since in the graded case many more different values must be predicted; moreover, in case the stimulus is used as ground truth, there is an inevitable discrepancy between the stimulus and the actual action performed by the subject. This increases the uncertainty. Notice that these error levels are obtained by training on one tenth of the available data, and are comparable to those presented in Castellini et al. (2012).

From these results we conclude that *a linear relationship exists between finger forces and ultrasound images.*

### 2.7. On-off training suffices to predict graded forces

#### 2.7.1. Aim

To check whether an on-off training suffices to accurately predict graded forces: if during training the system only sees data obtained while resting and applying maximum force, will it then be able to correctly predict intermediate force values?

#### 2.7.2. Results

**Figure 10:**
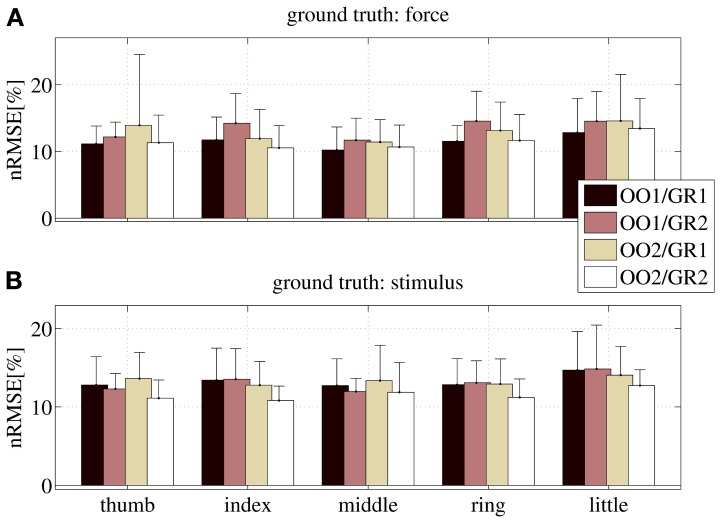
**nRMSE for all subjects, when training on an on-off phase and testing on a graded phase.** The legend denotes the training/testing phase, e.g., OO1/GR2 means that ridge regression was evaluated with data gathered during the first on-off phase, and the prediction was tested on data gathered during the second graded phase. **(A)** With the force as ground truth; **(B)** with the stimulus as ground truth. Each bar and stem represents the mean nRMSE and standard deviation obtained over all subjects.

Figure 10 shows the nRMSE obtained for all subjects, for each session (OO1, GR1, OO2 and GR2) and finger. In panel (Figure 10A) the force values are used as the ground truth, whereas in (Figure 10B) the stimulus values are used.

The overall nRMSE is clearly much larger than in the previous case, this time around 10% of the target range. Notice, however, that this error is remarkably consistent over subjects, fingers and chosen training/testing datasets (i.e., there is no statistically significant difference when OO1 or OO2 is used to estimate the linear regression, as well as there is no difference when testing on GR1 or on GR2). This error level is, again, comparable with that obtained in Castellini et al. (2012). Also, by comparing the upper and lower panels of Figure 10, it is apparent that there is no relevant difference when using the force as ground truth, or the stimulus.

From these results we conclude that *on-off training suffices to predict graded forces.*

## 3. Online implementation

As a last requirement, the system is expected to work inside a non-stationary environment, meaning that it must adapt to changing conditions; for example, the displacement of the ultrasound transducer due to a collision with an external object. In such a case, a non-incremental system would require a completely new training session from scratch to regain full functionality. As opposed to that, by exploiting the linearity of the relationship found in Section 2.6, we can extend the approach to incremental (or recursive) ridge regression. This approach enables us to add knowledge to the system at any point.

### 3.1. Incremental ridge regression

Consider again the ridge regression equation, **w** = *A***b** where, for the sake of simplicity, we have redefined *A* = (*X*^*T*^*X* + λ *I*_*d*_)^−1^ and **b** = *X*^*T*^**y**, and a new (sample,target) pair (**x**′,*y*′) acquired after **w** has been evaluated. The updated regression vector can be evaluated as **w**′ = *A*′**b**′, where *A*′ and **b**′ are obtained by juxtaposing the new sample to *X* and **y**:

$$X^{\prime }=\left[\begin{array}{c} X\\ x^{\prime } \end{array}\right]\text{ and }y^{\prime }=\left[\begin{array}{c} y\\ {}^{y^{\prime }} \end{array}\right]$$

Notice that, as expected, adding a new sample will not increase *d*, the size of the matrix *A*; notice as well, that there is no need to compute the inverse of *A*, since *A*′ and **b**′ can be directly evaluated, e.g., by using the Sherman-Morrison formula (Hager, 1989):

$$A^{\prime }=A-\frac{Ax^{\prime }{x^{\prime }}^{T}A}{1+{x^{\prime }}^{T}Ax^{\prime }}\text{ and }b^{\prime }=b+x^{\prime }y^{\prime }$$

With this approach the time complexity of updating the model is 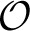
(*d*^2^), that is, independent of the total number of samples acquired so far, *n*.

### 3.2. Simulation of the online behavior

**Figure 11:**
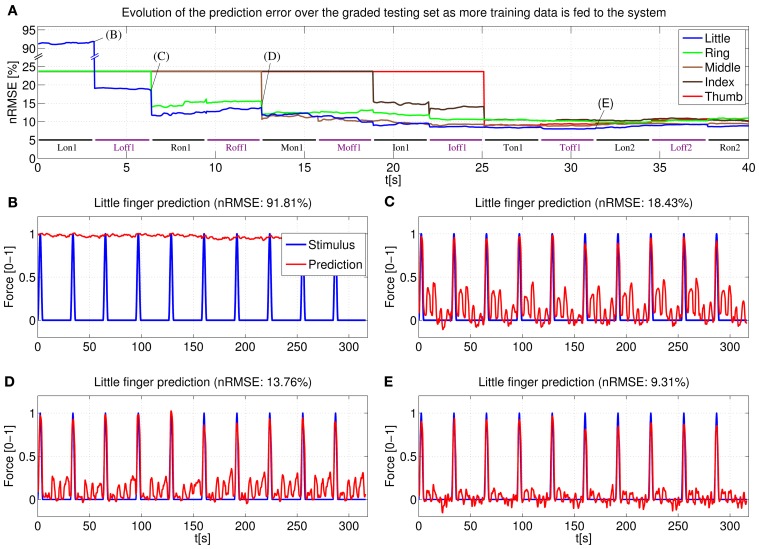
**System learning process for a typical subject. (A)** Evolution of the prediction error evaluated over the two graded sessions (GR1+GR2) as the system is fed on-off training data; **(B–E)** Little finger: stimulus target signal and force prediction at the training points B, C, D, and E (see **A**).

The behavior of an online system based upon the above stated remarks has been first simulated by “replaying” the (sample,target) pairs acquired during the main experiment of Section 2. Figure 11 shows the prediction error for a typical subject obtained by the online system. In particular, (Figure 11A) displays the error for each degree of freedom as the on-off training phase takes place: each time a new pair is acquired, the linear regression vector **w** is updated and the error for each finger is evaluated over the whole graded phase (GR1+GR2); (Figures 11B–E) show the prediction for one of the degrees of freedom (in this case, the little finger) after different sections of the on-off training phase have been completed.

Concretely, (Figure 11B) shows the force prediction of the little finger when the system has been trained only with the first on repetition of the little finger; in (Figure 11C) the system was trained with the first on repetition of the little finger and the first repetition of resting; in (Figure 11D) the system was trained with the first on repetition of the little finger, the first on repetition of the ring finger and the first two repetitions of resting; finally, in (Figure 11E) the system was trained with a complete on-off training round (that is, one on-off repetition for each finger, out of five—see Figure 4A, on-off part).

Notice the difference between the stimulus signal here and that of the graded part of Figure 4A. In this particular case we are only interested in the little finger, the space between each activation corresponds to the flexions of the other four fingers.

Consider now (Figure 4B). With the knowledge corresponding only to the maximum force of the little finger, the prediction cannot recognize any of the intermediate force values, or the resting position, and hence the 91.81% nRMSE (notice that the initial prediction error for the other fingers, as seen in (Figure 4A), is much lower. This is due to the skewed testing set, with predominant zero values. When the system has not seen any training information for a particular finger, it will predict always 0, which translates into a value of 23.63% NRMSE in the graded testing set).

The situation changes in (Figure 11C), where the system has already been trained with the maximum force of the little finger and the rest position; the prediction is accurate for all the intermediate force values of the little finger; however, since the knowledge of the system is limited only to the little finger, the prediction reacts to the flexions of the other fingers and this cross-talk causes a 18.43% nRMSE. In (Figure 11D) the system has already *seen* what the features for the ring finger look like, and so the little finger force prediction does not react when the ring finger is exerting force (notice that the ring finger flexion comes right after the little finger flexion), lowering the error to 13.76%. Lastly, once a complete on-off training round has been completed, the little finger force prediction is still accurate over the whole range of force values while keeping “silent” when the other fingers are exerting force. Further on-off rounds have no effect on the prediction, obtaining a relatively flat prediction error after the first training round.

### 3.3. Implementation

We have integrated the aforementioned algorithm into a C# software application. All the necessary algebraic operations during training (including the update of the inverse matrix with the Sherman-Morrison formula) require an average of 16.5ms; in contrast, producing a prediction during the prediction mode requires only an average of 3.7ms. Considering that gathering a new valid frame requires an average of 33.9 ms (29.5 Hz) and that both operations are computed in parallel in a multi-threaded environment, training or predicting does not affect the global frequency of the system and no frames are lost without processing.

Obviously, since the training can happen at each new frame, the system can be switched from training to prediction mode and vice versa at any point and without losing any previous knowledge. This allows us to adjust the training length to the strictly necessary. Should the prediction not have the desired accuracy or become worse after a perturbation, it is possible to go back to training and give the system more knowledge about the desired finger/fingers.

**Figure 12:**
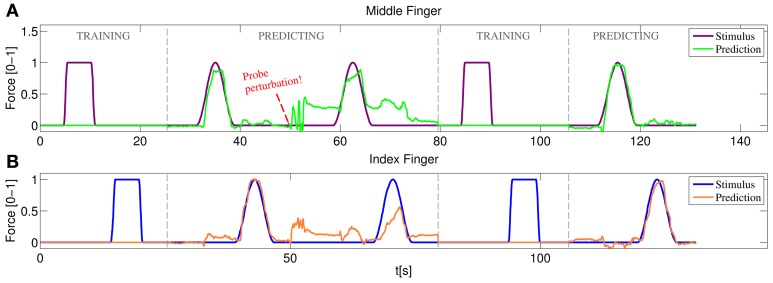
**Online training/testing for a typical subject.** After a perturbation in the position of the US probe reduces the quality of the prediction, the system is set again to training and more information is fed to the system. After a fast retraining phase the prediction recovers the accuracy obtained with the initial training. **(A)** Stimulus and prediction values for the middle finger; **(B)** stimulus and prediction values for the index finger.

Figure 12 represents the training/testing process for a typical subject. In this case the middle and index fingers were trained, both with only one on-off repetition. Once the training was completed the prediction mode was enabled. A sinusoidal stimulus was launched for each finger and the subject was asked to follow it as closely as possible (basically the subject had to apply force so that the 3D hand model displaying the prediction moved exactly as the model displaying the stimulus). As can be seen in the figure, starting at approximately 32 s for the middle finger and at 38 s for the index finger, both the sinusoidal stimulus and the prediction look remarkably similar. Concretely, during this sinusoidal stimulus a nRMSE prediction error of 6.07% and 8.52% was obtained for index and middle finger, respectively.

Soon after, the ultrasound transducer was manually shifted and then placed at approximately the same position it was in the beginning. The subject was asked again to follow a sinusoidal stimulus, failing to do so and obtaining 22.77% and 24.01% nRMSE prediction errors. A new training round was then launched, again with only one on-off repetition per finger. Back to the prediction mode, the subject was able, once again, to replicate accurately the movement of the stimulus hand reducing the errors to 6.27% and 6.86% for index and middle finger respectively.

For a live demonstration of the online system, please refer to the movie in the Supplementary material. In the movie, the stimulus and prediction are displayed using two separate Blender 3D hand models. The force of the fingers is proportional to their flexion angle in the model and maximum force is represented by a completely flexed finger (as during the on-off training).

## 4. Discussion

In previous work (Jing-Yi et al., 2011; Castellini et al., 2012) it has been shown that medical ultrasound imaging has great potential as a novel human-machine interface, with the main application of controlling an advanced hand prosthesis. In the current work we have pushed the envelope to the point that we now claim that US imaging is mature to be delivered to the clinics, as part of a new form of treatment. In particular, the results shown here indicate that it works in a realistic scenario, that is, it is practically usable by disabled subjects.

### 4.1. A realistic scenario for US imaging as an HMI

Too many a time a human-machine interface is studied with little or no reference to its practical application. In this work we have tried to give a sensible definition of “realistic scenario” for US imaging to be used, e.g., by amputees, and we have tested whether it would deliver good results^2^. In particular, amputees require at least

1. that the training (calibration) phase be short;
2. that the calibration entail simple imitation tasks;
3. that it need no sensors; and lastly,
4. that the system be able to acquire new knowledge when required.

The first three items are motivated by (1) the generally bad condition of a stump, which quickly elicits fatigue and stress; (2) the lack of sensory feedback from the missing limb, which makes it hard (if not impossible) for the amputee to apply graded forces; (3) the absence, *in principle*, of ground truth coming, e.g., from force sensors and/or datagloves. The fourth item is motivated, first and foremost, by the necessity of retraining previous patterns in case the signal changes due to, e.g., movement of the ultrasound transducer, or in order to improve the current prediction in case the subject is unsatisfied with it; as well, it is motivated by the desire by the subject to learn new patterns, if required. Regarding item (2), notice that the vast majority of amputees have phantom feelings that *do not* correspond to the intended force/movement patterns; therefore a further effort is required to ignore the feeling and this further motivates the requirement for a simple calibration task.

The realistic scenario we have set up consists of an experimental protocol showing that, on intact subjects, US imaging works exactly according to the above four requirements. In particular, the results of Section 2.6 show that a linear relationship exists between simple first-order spatial features extracted from the US images, and fingertip forces; and that the same relationship is found when the visual stimulus is used as ground truth, both when on-off data is employed, and in presence of graded-force tasks. Moreover, in Section 2.7 we show that training on the on-off data suffices to predict graded forces, both when the force sensor data is used as ground truth, and when the visual stimulus is. Lastly, Section 3 shows that, by exploiting the linearity of the relationship described in Section 2.6, an online system can be built, based upon incremental ridge regression, able to predict finger forces incrementally; it works in cinema-quality real-time (30 Hz) *both during training and during prediction*, and it can be seamlessly switched from prediction to training, enabling corrections or new patterns to be learned on-the-fly.

This last characteristic is particularly important in case the probe moves with respect to the subject's skin, or in case the subject assumes a very different posture with respect to the one she had kept during training. In both cases the ultrasound image may substantially change from what it was during training, and since we enforce no mechanism to compensate the probe movement, new data must be acquired to restore the prediction accuracy. There is many a reason for choosing this alternative way, as opposed to the compensation mechanism based on optical flow enforced in Castellini et al. (2012). Firstly, in normal conditions the probe essentially does not shift, thanks to the fixing cradle (Figures 2C,D); the problem appears only in extreme cases - see the movie in the Supplemental Material: the experimenter must manually shift it in order to cause a disruption in the prediction. Secondly, in an initial round of experiments, the optical-flow-based mechanism did not yield good results applied in this setting; we speculate that this is due to the intrinsically complex nature of the image deformations that appear in the ultrasound setting. Moreover, computing the optical flow in order to shift the interest points around requires one or more reference frames, and can be computationally hard to evaluate (Horn and Schunk, 1981). Lastly, retraining, in our system, is extremely fast and accurate, and represents a valid alternative approach as we have demonstrated.

We believe that this last point is particularly important, and could be of help in any pattern-matching-based approach to HMIs. If retraining is affordable (i.e., fast and accurate), then it can be used to compensate for any shift in the input probability distribution, be it of physical, physiological or any other nature.

### 4.2. Applications and future directions

Hand amputees probably constitute only one of the possible patient communities who could benefit from the use of this novel HMI. Ultrasound imaging machines cannot, at the current state of the art, be miniaturized to the extent of being embedded in a prosthesis, and this rules out its use as a wearable control system for a hand prosthesis, although a hand-held ultrasound machine could be easily carried by the patient in a bag. More realistically, such a system could be used to control a robotic wheelchair; or even employed in a hospital in a non-portable form, to provide a novel treatment against neuropathic pain. One could think of this system as a way of visualizing the imaginary limb of impaired subjects such as, besides amputees, patients of complex regional pain syndrome and nerve/muscle impairments. A lesser form of such a therapy already exists, it is called mirror therapy and stems from Ramachandran's seminal discovery (Ramachandran et al., 1995) that a visual illusion of the missing hand can alleviate phantom pain [see also, for a more recent result along this line, Chan et al. (2007)].

The application of this potential therapy to more severe amputees (above-elbow or even disarticulated at the shoulder) is as well a fascinating possibility. First of all, ultrasound imaging could be used to reconstruct the intended movements/torques/forces at the elbow and shoulder, that is, patterns which are most likely still present in the stump of such severly mutilated patients. Interestingly however, research by Mercier, Reilly, Sirigu and others (Mercier et al., 2006; Reilly et al., 2006) has shown that stable electromyographic patterns referring to, e.g., the thumb opening and closing still exist in above-elbow amputees—patients in which the related muscular structure is not present any longer. This phenomenon is explained as the result of after-trauma spontaneous reinnervation appearing at the local level. Ultrasound imaging is probably accurate enough to detect those patterns and could therefore be used to visualize the imaginary hand in such cases, too.

Currently, the system employs 181 interest points and extracts 543 features from them; an ongoing study has however revealed that this number can be dramatically reduced without any apparent degradation in the performance. We are in the process of applying this new features extraction schema in the online version. On an even more interesting side, testing the system on a selected pool of amputees is planned as the very next step; in that case some form of visual-feedback method to convey the right patterns from the subjects will probably be required.

## 5. Conclusion

In conclusion, this work describes a realistic implementation of medical ultrasound imaging as a novel human-machine interface for the disabled. We show that ultrasound images of the forearm, obtained from a standard ultrasound machine, can be used to quickly and reliably visualize the forces required at the fingertips. This can be done in real-time, incrementally, and employing very simple tasks for training, as amputees or other neuropathic pain patients would be able to do. We speculate that US imaging has therefore the potential to become the basis of a treatment for neuropathic pain, be it phantom-limb pain or consequent nerve/muscle injuries.

### Conflict of interest statement

The authors declare that the research was conducted in the absence of any commercial or financial relationships that could be construed as a potential conflict of interest.

## Footnotes

^1^The reader should not be deceived by the Euclidean distances visible in Figure 6, which are much smaller than those obtained in the non-reduced input space of dimension 543.

^2^We claim that most of the considerations about a realistic implementation of the US-based HMI would hold for any other HMI whatsoever to be employed in this field.
